# Adiponectin Gene Polymorphisms and Acute Respiratory Distress Syndrome Susceptibility and Mortality

**DOI:** 10.1371/journal.pone.0089170

**Published:** 2014-02-19

**Authors:** Amy M. Ahasic, Yang Zhao, Li Su, Chau-Chyun Sheu, B. Taylor Thompson, David C. Christiani

**Affiliations:** 1 Section of Pulmonary, Critical Care and Sleep Medicine, Department of Medicine, Yale School of Medicine, New Haven, Connecticut, United States of America; 2 Department of Epidemiology and Biostatistics, School of Public Health, Nanjing Medical University, Nanjing, China; 3 Environmental and Occupational Medicine and Epidemiology Program, Department of Environmental Health, Harvard School of Public Health, Boston, Massachusetts, United States of America; 4 Division of Pulmonary and Critical Care Medicine, Kaohsiung Medical University Hospital, Kaohsiung Medical University, Kaohsiung, Taiwan; 5 Pulmonary and Critical Care Unit, Department of Medicine, Massachusetts General Hospital, Boston, Massachusetts, United States of America; MOE Key Laboratory of Environment and Health, School of Public Health, Tongji Medical College, Huazhong University of Science and Technology, China

## Abstract

**Rationale:**

Adiponectin is an anti-inflammatory adipokine that is the most abundant gene product of adipose tissue. Lower levels have been observed in obesity, insulin resistance, and in critical illness. However, elevated levels early in acute respiratory failure have been associated with mortality. Polymorphisms in adiponectin-related genes (*ADIPOQ*, *ADIPOR1*, *ADIPOR2*) have been examined for relationships with obesity, insulin resistance and diabetes, cardiovascular disease, and to circulating adipokine levels, but many gaps in knowledge remain. The current study aims to assess the association between potentially functional polymorphisms in adiponectin-related genes with acute respiratory distress syndrome (ARDS) risk and mortality.

**Methods:**

Consecutive patients with risk factors for ARDS admitted to the ICU were enrolled and followed prospectively for development of ARDS. ARDS cases were followed through day 60 for all-cause mortality. 2067 patients were successfully genotyped using the Illumina CVD BeadChip high-density platform. Of these, 567 patients developed ARDS. Forty-four single nucleotide polymorphisms (SNPs) on *ADIPOQ*, *ADIPOR1* and *ADIPOR2* were successfully genotyped. Of these, 9 SNPs were hypothesized to be functional based on their location (promoter, exon, or 3′ untranslated region). These 9 SNPs were analyzed for association with ARDS case status and mortality among ARDS cases.

**Results:**

After multivariable analysis and adjustment for multiple comparisons, no SNPs were significantly associated with ARDS case status. Among ARDS cases, homozygotes for the minor allele of rs2082940 (*ADIPOQ*) had increased mortality (hazard ratio 2.61, 95% confidence interval 1.36–5.00, *p* = 0.0039) after adjustment for significant covariates. The significance of this association persisted after adjustment for multiple comparisons (*FDR_q* = 0.029).

**Conclusions:**

A common and potentially functional polymorphism in *ADIPOQ* may impact survival in ARDS. Further studies are required to replicate these results and to correlate genotype with circulating adiponectin levels.

## Introduction

Adipose tissue is now recognized as an active endocrine organ. Adipocytes, the primary cells making up adipose, secrete adipokines that have been studied as phenotypic markers for obesity. Adipokines are involved in vascular and generalized inflammation, as well as insulin resistance and atherogenesis [1]–[5]. They have also been studied in animal models of sepsis [6], [7].

Adiponectin is an anti-inflammatory, anti-diabetic, and anti-atherogenic adipokine that is the most abundant gene product of adipose tissue [1]–[4], [8]. Adiponectin is encoded by *ADIPOQ*, located on chr3q27. Adiponectin binds to the AdipoR1 and AdipoR2 receptors, encoded by *ADIPOR1* (chr1q32) and *ADIPOR2* (chr12p13) respectively. AdipoR1 is ubiquitously expressed, particularly in skeletal muscle, while AdipoR2 is primarily expressed in liver as well as skeletal muscle [9], [10]. These receptors mediate the biologic effects of adiponectin, and they have strong similarities to G-protein-coupled receptors although they are distinct from this class of receptors. Binding of adiponectin to its receptors results in activation of unique signaling molecules such as AMPK, p38 MAPK, and PPAR-α [10].

Adiponectin has been studied widely in obesity and diabetes, and it is a key suppressor of the metabolic derangements leading to insulin resistance, type 2 diabetes mellitus, metabolic syndrome, and cardiovascular disease [11]. For instance, lower plasma levels of plasma adiponectin have been observed in obesity, insulin resistance, and type 2 diabetes mellitus [12]–[15]. Adiponectin is also inversely correlated with body mass index (BMI) [9], [16]. Genetic studies have investigated the association between adiponectin-related genes and a wide variety of health outcomes, including obesity, diabetes, cardio- and cerebrovascular disease and dyslipidemia, nonalcoholic fatty liver disease, and a variety of cancers including breast, colon and prostate [17].

Adiponectin acts through multiple pathways: it enhances glucose utilization through AMPK phosphorylation; it antagonizes TNF-alpha by negatively regulating its expression in various tissues such as liver and macrophages; and it inhibits endothelial NF-kappa-B signaling through a cAMP-dependent pathway [1], [2], [4]. It may exert its anti-inflammatory effects by limiting aberrant leukocyte-endothelial interactions and augmenting nitric oxide availability [16]. Lower adiponectin levels have been associated with inflammation, oxidative stress, and glucocorticoids, and the link with insulin signaling suggests that adiponectin secretion is highly affected by the insulin sensitivity of adipocytes [1]. It has been postulated that adiponectin may be associated with development of insulin resistance during critical illness [18].

Because of its links to inflammation and insulin sensitivity, adiponectin has been investigated in a few critical care studies in humans [16], [18]–[21]. In general, these studies observed lower levels of adiponectin at the onset of critical illness, with a rise during recovery [16], [18], [19], [21]. In one study of acute respiratory failure patients, higher levels of adiponectin were associated with fewer ventilator-free days and higher mortality [16]. This limited literature on adiponectin in critical illness has not included studies of genetic variations of the genes encoding adiponectin and its receptors. Thus, the current study aims to investigate the association between genetic variations in adiponectin-related genes and the development of and survival in acute respiratory distress syndrome (ARDS) in a large intensive care unit (ICU) cohort.

## Materials and Methods

The Human Subjects Committees (HSC) at the Massachusetts General Hospital (MGH) and the Harvard School of Public Health approved this study, and all participants or appropriate surrogates provided informed consent and signed HSC-approved consent forms.

### Study Population and Design

This study is part of the ongoing Molecular Epidemiology of ARDS Study of which study design and exclusion criteria have been described in depth previously [22]. Adult ICU admissions to MGH (Boston, MA) from January 1999 to March 2009, and at Beth Israel-Deaconess Medical Center (BIDMC, Boston, MA) from January 2007 to February 2009 were screened daily for study-defined clinical risk factors for ARDS: pneumonia, sepsis or septic shock, aspiration, massive transfusions, pulmonary contusion or multiple fractures. Eligible patients were approached and enrolled in the prospective cohort after consent was obtained. Patients were followed daily until ICU discharge or death. They were defined as having ARDS if they developed respiratory failure requiring intubation and mechanical ventilation, and if they met the North American-European Consensus Conference (AECC) criteria for ARDS: arterial oxygen tension/fraction of inspired oxygen ratio (PaO_2_/FiO_2_) of ≤200 mm Hg; bilateral infiltrates on chest radiograph; and pulmonary arterial occlusion pressure ≤18 mm Hg, or no clinical evidence of left atrial hypertension. In the parent study, ARDS patients are cases, and patients with ARDS risk factors who did not develop ARDS are controls. Day 0 of ARDS was defined as the day on which the case patient first met all AECC criteria simultaneously. ARDS cases were followed until Day 60 for all-cause mortality; controls were not followed for mortality. The primary outcomes of the current substudy are (1) development of ARDS in the at-risk cohort, and (2) death among ARDS cases.

### Genotyping

Genotyping of the parent cohort has been described in detail previously [23], [24]. Briefly, genomic DNA was extracted from whole blood drawn during the first 48 hours of admission using the Autopure LS robotic workstation (Gentra Systems, Minneapolis, MN) and DNA Purification Reagent Kits (Qiagen, Valencia, CA). Genotyping was done at the Center for Applied Genomics, Children's Hospital of Philadelphia (Philadelphia, PA), using the Illumina Human-CVD BeadChip (Illumina, San Diego, CA). The Human-CVD BeadChip is a high-density genotyping platform covering 48,742 markers across roughly 2100 genes associated with cardiovascular, metabolic, and inflammatory pathways [25]. Genotyping personnel were blinded to ARDS case status, and samples were processed according to Illumina's protocols. Quality control has also been described previously [23], [24].

Forty-four single nucleotide polymorphisms (SNPs) on *ADIPOQ*, *ADIPOR1* and *ADIPOR2* are included in the CVD BeadChip platform. Of these, we hypothesized 9 SNPs *a priori* as functional based on location, and as common with MAF>5% (Table 1). These 9 SNPs were then analyzed for association with ARDS case status, and mortality among ARDS cases.

**Table 1:** Selected Adiponectin and Receptor SNPs and Genotype Frequencies in the Total Cohort.

			Genotype, N (%)			
Gene	Polymorphism	Location	AA*	AB	BB	MAF**
*ADIPOQ*	rs266729	Promoter	1151 (56)	790 (38)	120 (6)	25.0%
*ADIPOQ*	rs822387	5′ of Promoter	1699 (82)	349 (17)	17 (1)	9.3%
*ADIPOQ*	rs864265	5′ of Promoter	1531 (74)	496 (24)	40 (2)	13.3%
*ADIPOQ*	rs2082940	3′ UTR	1562 (76)	453 (22)	49 (2)	13.4%
*ADIPOQ*	rs16861194	Promoter	1738 (84)	496 (24)	40 (2)	8.3%
*ADIPOR1*	rs7539542	3′ UTR	929 (45)	914 (44)	224 (11)	32.9%
*ADIPOR2*	rs1029629	5′ of Promoter	999 (48)	865 (42)	203 (10)	30.7%
*ADIPOR2*	rs1044471	3′ UTR	536 (26)	1076 (52)	455 (22)	48.1%
*ADIPOR2*	rs16928751	Coding exon	1626 (79)	409 (20)	30 (1)	11.4%

*A denotes major allele; B denotes minor allele;

**MAF = minor allele frequency.

### Statistical Analysis

Demographic and clinical characteristics between various groups were compared using chi-square tests for categorical variables, and by Wilcoxon tests for continuous variables. Correlations between genotypes and BMI or diabetes were tested by calculating Spearman correlation coefficients. Diabetes status was abstracted from the medical record with any physician-recorded documentation of type 1 or type 2 diabetes mellitus defining positive diabetes status.

Logistic regression models were used to test the association of SNP genotypes with ARDS case status. Cox proportional hazards models were used to assess the association between SNP genotypes and mortality. A multivariable model for each outcome without genotypes was used to select significant predictors. A backward elimination algorithm with *p*≤0.2 for entry into the model was used to test significance of the following clinically relevant covariates: age, gender, Acute Physiology and Chronic Health Evaluation (APACHE) III score, risk factor for ARDS (eg. sepsis, pneumonia, trauma), massive transfusion (defined as requiring ≥8 units of red blood cells in 24 hours), and current history of cirrhosis. Diabetes status and BMI were also included *a priori* given the known biology of adiponectin. In multivariable analyses, the APACHE III score was revised to exclude age and PaO_2_/FiO_2_ to avoid collinearity [26]. BMI was considered as a 4-category variable using the National Heart, Lung, and Blood Institute's cutoffs for underweight, normal weight, overweight, and obese (<18.5, 18.5–24.9, 25–29.9, and ≥30 respectively) [27]. The significant variables were then used in multivariable models that included genotypes for each SNP. Given no preexisting knowledge of mode of inheritance, additive, recessive, and dominant modes were considered for all SNPs. Correction for multiple comparisons was done using positive false discovery rate (FDR) statistics. The *FDR_q* values were calculated using multivariable models with the significant variables as above. All statistical analyses were performed using SAS Version 9.3 (SAS Inc., Cary, North Carolina).

## Results

### Patient population

From September 1999 to April 2009, consecutive ICU admissions were screened for possible inclusion. In total, 4244 patients were eligible, of whom 2786 patients were successfully consented, enrolled and followed for development of ARDS. Of those enrolled, 387 had no or poor quality DNA samples, and an additional 101 patients had genotyping call rates ≤95%. Also excluded were 22 patients with incomplete clinical data, previous enrollment, or previous history of ARDS, and 209 patients who were not of European descent. Racial and ethnic categorization was abstracted from hospital registration information with self-report from patients or their surrogates. No genetic determination of ancestry was attempted in this cohort. Thus, 2067 white patients were successfully genotyped using the BeadChip at the loci of interest. Of these, 567 patients developed ARDS.

Cases and control patients differed significantly in age, APACHE III, and sepsis, septic shock, or direct pulmonary injury as the risk factor ARDS (Table 2). Among ARDS cases, survivors and nonsurvivors also differed significantly in age, APACHE III, and septic shock or trauma as the risk factor ARDS (Table 3). BMI was also significantly higher in ARDS cases than controls, although BMI among survivors with ARDS was higher than in nonsurvivors (Tables 2 and 3). Of note, the median for all groups fell in the overweight range.

**Table 2:** Baseline Cohort Characteristics.

	Total Cohort (n = 2067)	Controls (n = 1500)	Cases (n = 567)	*P* *
Age, mean ± SD	61.5±17.4	62.5±17.1	58.8±18.0	<0.0001
Female gender, n (%)	785 (38)	577 (38)	208 (37)	0.46
BMI, median (IQR)	26.5 (8.0)	26.4 (7.8)	27.4 (9.0)	0.017
APACHE III score, mean ± SD	60.4±21.5	58.7±20.9	65.3±22.2	<0.0001
Sepsis syndrome, n (%)	644 (31)	510 (34)	134 (24)	<0.0001
Pulmonary source	363 (56)	260 (51)	103 (77)	
Septic shock, n (%)	1035 (50)	672 (45)	363 (64)	<0.0001
Pulmonary source	561 (54)	302 (45)	259 (71)	
Trauma, n (%)	174 (8)	130 (9)	44 (8)	0.51
Massive transfusion, n (%)	215 (10)	161 (11)	54 (10)	0.42
Aspiration, n (%)	143 (7)	95 (6)	48 (9)	0.09
Direct pulmonary injury, n (%)	1153 (56)	730 (49)	423 (75)	<0.0001

*P-value reflects comparison between cases and controls.

SD = standard deviation; IQR = interquartile range; NS = not significant.

**Table 3:** Cohort Characteristics of ARDS Cases by Mortality.

	Survivors (n = 355)	Nonsurvivors (n = 212)	*P* *
Age, mean ± SD	54.5±18.1	66.1±15.4	<0.0001
Female gender, n (%)	124 (35)	84 (40)	0.26
BMI, median (IQR)	27.8 (9.7)	26.1 (6.8)	0.005
APACHE III score, mean ± SD	58.1±19.6	77.5±20.9	<0.0001
Sepsis syndrome, n (%)	88 (25)	46 (22)	0.40
Pulmonary source	65 (74)	38 (83)	
Septic shock, n (%)	214 (60)	149 (70)	0.02
Pulmonary source	161 (75)	98 (66)	
Trauma, n (%)	41 (12)	3 (1)	<0.0001
Massive transfusion, n (%)	35 (10)	19 (9)	0.72
Aspiration, n (%)	28 (8)	20 (9)	0.52
Direct pulmonary injury, n (%)	267 (75)	156 (74)	0.67

*P-value reflects comparison between survivors and nonsurvivors.

SD = standard deviation; IQR = interquartile range; NS = not significant.

### Genotype analysis

Genotype frequencies in this cohort for the 9 polymorphisms from *ADIPOQ*, *ADIPOR1* and *ADIPOR2* are displayed in Table 1. Our genotype frequencies are similar to publicly available databases of European samples. Overall genotyping success rate was 99.9% among the 2067 successfully genotyped with the chip.

Spearman correlation coefficients were calculated for genotype at each SNP by BMI and by diabetes status. There were no significant correlations between BMI and any SNP. There was a statistically significant but weak positive correlation between rs16861194 genotype (increasing number of variant alleles from 0 to 2) and diabetes status (ρ = 0.060, *p* = 0.0068).

### Significant predictors of primary outcomes

As described above, multivariable logistic regression and Cox proportional hazards regression were used to select relevant covariates for inclusion in models with genotypes, with a conservative p<0.2 for inclusion in the model. Significant predictors for development of ARDS were: age [odds ratio (OR) 0.98, 95% confidence interval (CI) 0.98–0.99, *p*<0.0001]; BMI (OR 1.20, 95% CI 1.08–1.33, *p* = 0.0005); septic shock (OR 1.86, 95% CI 1.50–2.32, *p*<0.0001); direct pulmonary injury (OR 3.64, 95% CI 2.92–4.54, *p*<0.0001); diabetes (OR 0.56, 95% CI 0.44–0.73, *p*<0.0001); and need for RBC transfusions (OR 1.57, 95% CI 1.28–1.94, *p*<0.0001). Among ARDS cases, significant predictors of mortality were: age [hazard ratio (HR) 1.03, 95% CI 1.02–1.04, *p*<0.0001]; BMI (HR 0.89, 95% CI 0.79–1.01, *p* = 0.075); cirrhosis (HR 2.40, 95% CI 1.54–3.72, *p*<0.0001); APACHE III (HR 1.02, 95% CI 1.02–1.03, *p*<0.0001); and trauma (HR 0.21, 95% CI 0.067–0.67, *p*<0.0078). As mentioned, BMI and diabetes status were chosen *a priori* to be included in all models based on known biology of adiponectin.

### Adiponectin-related genes and development of ARDS

The presence of the variant allele of rs1029629 on *ADIPOR2* was significantly associated with development of ARDS in the unadjusted model (OR 0.81, 95% CI 0.66–0.98, *p* = 0.03). This did not persist, however, after adjustment for significant covariates (*p* = 0.05), and adjustment for multiple testing (*FDR_q* = 0.20). The presence of the variant allele of rs16928571, also on *ADIPOR2*, was associated with development of ARDS in both unadjusted and multivariable models (OR 1.28, 95% CI 1.00–1.64, p = 0.05; OR 1.39, 95% CI 1.06–1.83, p = 0.02). When adjusting for multiple comparisons, however, the result was not significant (*FDR_q* = 0.13). No other SNPs showed any significant association with development of ARDS.

### Adiponectin-related genes and mortality among ARDS cases

Genotype frequencies in ARDS cases, including those in survivors and nonsurvivors, are shown in Table 4. In unadjusted analysis, homozygous variants of rs2082940 on *ADIPOQ* had a highly significant increase in mortality (Table 5). This association remained significant after adjusting for both relevant covariates and for multiple comparisons. Hazard ratios for the other variables included in the model are as follows: age (HR 1.03, 95% CI 1.02–1.04, *p*<0.0001); APACHE III (HR 1.02, 95% CI 1.01–1.03, *p*<0.0001); BMI (HR 0.87, 95% CI 0.75–1.00, *p* = 0.05); cirrhosis (HR 2.37, 95% CI 1.48–3.81, *p* = 0.0004); diabetes (HR 1.19, 95% CI 0.83–1.69, *p* = 0.34); and trauma (HR 0.25, 95% CI 0.079–0.79, *p* = 0.018). No other SNPs were associated with mortality in the ARDS cases. Results were similar when modeling survival using logistic regression.

**Table 4:** Genotype Frequencies in ARDS Cases.

	ARDS Cases, N (%)			Survivors N (%)			Nonsurvivors N (%)		
	AA*	AB	BB	AA	AB	BB	AA	AB	BB
rs266729	312 (55)	220 (39)	33 (6)	192 (54)	138 (39)	25 (7)	120 (57)	82 (39)	8 (4)
rs822387	464 (82)	99 (17)	4 (1)	292 (82)	61 (17)	2 (1)	172 (81)	38 (18)	2 (1)
rs864265	422 (74)	137 (24)	8 (2)	267 (75)	81 (23)	7 (2)	155 (73)	56 (26)	1 (1)
rs2082940	435 (77)	120 (21)	11 (2)	285 (80)	69 (20)	1 (<1)	150 (71)	51 (24)	10 (5)
rs16861194	477 (84)	88 (16)	2 (<1)	302 (85)	51 (14)	2 (1)	175 (83)	37 (17)	0 (0)
rs7539542	247 (44)	260 (46)	60 (11)	159 (45)	161 (45)	35 (10)	88 (42)	99 (47)	25 (12)
rs1029629	252 (44)	257 (45)	58 (10)	159 (45)	157 (44)	39 (11)	93 (44)	100 (47)	19 (9)
rs1044471	157 (28)	276 (49)	134 (24)	102 (29)	167 (47)	86 (24)	55 (26)	109 (51)	48 (23)
rs16928751	463 (82)	95 (17)	9 (1)	286 (80)	66 (19)	3 (1)	177 (83)	29 (14)	6 (3)

*A denotes major allele; B denotes minor allele.

**Table 5:** Cox Proportional Hazards Models of Mortality by Genotype in ARDS Cases (n = 566).±

	Crude HR (95% CI)	*P*	Adjusted HR*(95% CI)	*P*	*FDR_q*
***ADIPOQ***					
*rs266729*	0.60 (0.30–1.21)	0.16	0.71 (0.34–1.45)	0.34	0.37
*rs822387*	1.07 (0.27–4.30)	0.93	1.95 (0.48–7.91)	0.35	0.37
*rs864265*	0.27 (0.038–1.92)	0.19	0.23 (0.032–1.64)	0.14	0.27
*rs2082940*	4.09 (2.16–7.74)	<0.0001	2.61 (1.36–5.00)	0.004	0.02
*rs16861194*	—**	0.97	—**	0.98	0.68
***ADIPOR1***					
*rs7539542*	1.18 (0.78–1.79)	0.44	1.46 (0.94–2.27)	0.09	0.27
***ADIPOR2***					
*rs1029629*	0.87 (0.54–1.39)	0.56	0.96 (0.59–1.57)	0.87	0.68
*rs1044471*	0.98 (0.71–1.36)	0.91	0.92 (0.66–1.29)	0.64	0.57
*rs16928751*	2.12 (0.94–4.78)	0.07	1.78 (0.77–4.09)	0.17	0.27

± Models shown represent recessive inheritance pattern.

*Also included in the model: age, BMI, APACHE, trauma, cirrhosis, diabetes.

**HR could not be calculated because only 0.7% of subjects genotyped at this locus had the homozygous variant genotype.

HR = hazard ratio; CI = confidence interval; *FDR_q* = false discovery rate q-value.

**Figure 1:**
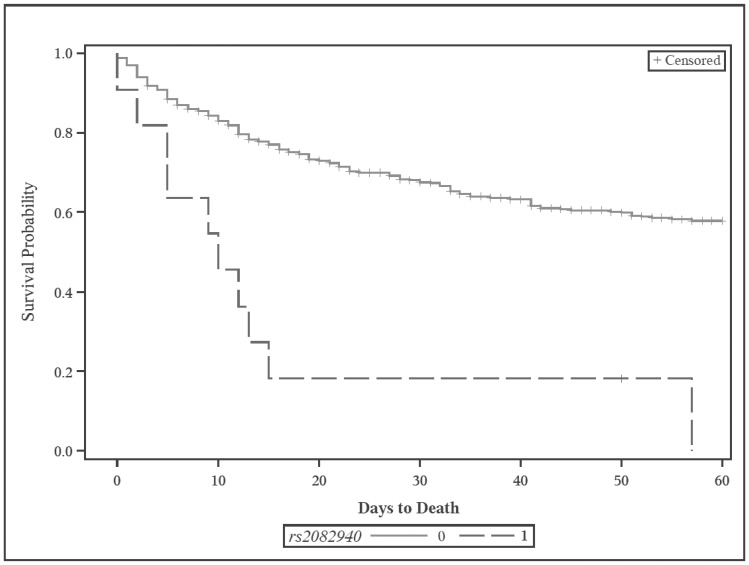
Kaplan-Meier survival plot for rs2082940 among ARDS case patients. All case patients were followed out to 60 days from enrollment. Note: 0 = non-homozygous variant genoypte; 1 = homozygous variant genotype.

Among the 11 homozygous variants of rs2082940, 9 of the 10 nonsurivors died between 0 and 15 days after ARDS onset, and one patient died after a prolonged hospital and ICU course, 57 days after onset of ARDS. The one survivor also had a protracted hospital and ICU course. Among all 11 patients, there were no major outliers in basic demographics or clinical features. Of note, however, 9 patients were overweight or obese, and 10 had sepsis or septic shock, higher percentages than in our total cohort. The sole survivor was also the only underweight patient of the 11, with an ICU admission BMI of 18.0. An unadjusted Kaplan-Meier survival plot is shown in Figure 1.

## Discussion

This is the first study in the literature to report on variants of *ADIPOQ*, *ADIPOR1* and *ADIPOR2* in association with mortality in critically ill adults. Specifically, our results show a significant increase in mortality for ARDS patients homozygous for the rs2082940 variant. The rs2082940 polymorphism has also been associated with increased risk of developing Type 2 diabetes mellitus [28] and decreased risk of prostate cancer [29]. This SNP is common (MAF 13.4%) and is located in the 3′ UTR of *ADIPOQ*. It has been shown in other studies to affect circulating levels of adiponectin with the variant allele associated with increased levels of adiponectin [28], [29]. Thus, inference would suggest that the higher mortality seen in our ARDS patients may be associated with higher levels of adiponectin. Although we do not have corresponding plasma adiponectin measurements in this cohort, this inference would be consistent with previous human studies showing lower adiponectin levels at ICU admission are associated with improved survival [16], [20].

Having said this, we know from previous genetic studies that while adiponectin levels are highly heritable, SNPs in *ADIPOQ* account for only a small percent of variability in circulating adiponectin, ranging from <2% to 8% across studies [30]–[32]. Thus, there are likely other important factors such as epigenetic and environmental factors, interactions between loci, copy number variations, and genetic polymorphisms outside of *ADIPOQ* [30], [31]. For instance, the GEMS study identified a locus within *CDH13* that was strongly associated with adiponectin levels [31]. This was reiterated in a genome-wide association study (GWAS) of adiponectin levels in Korean adults in which polymorphisms in the cadherin 13 gene (*CDH13*) made up 6 of the top 20 hits, and *ADIPOQ* polymorphisms were not even represented among the top 20 [33]. This may also explain why we did not see any significant results for rs822387, a polymorphism in the *ADIPOQ* promoter that has been significantly associated with plasma adiponectin levels in several studies [30], [32]. This finding was not replicated, however, in a whole genome analysis within the GEMS study [31], or in a multiethnic meta-analysis [34]. Thus the functions of *ADIPOQ* clearly go beyond just expression of adiponectin.

There have also been several previous genome-wide association studies that have identified expression quantitative trait loci (eQTLs) for plasma adiponectin [30], [31], [34]. These include rs6810075, rs17300539/rs822387, rs17366568, and rs6773957. Using the SNP Annotation and Proxy Search database (SNAP, http://www.broadinstitute.org/mpg/snap/), pairwise linkage analysis reveals that both rs6773957 and rs17366568 are in complete linkage disequilibrium with rs2082940 (D′ = 1.0) [35]. However, r^2^ for both eQTLs is low (0.19 and 0.02 respectively), likely reflecting differences in allele (and thus haplotype) frequencies. Still, the high D′ suggests a strong coinheritance of rs2082940 with these eQTLs. In pairwise analysis with the remaining eQTLs and rs2082940, linkage disequilibrium was extremely low (D′≤0.007).

Since adiponectin is anti-inflammatory in nature, it is somewhat unintuitive that lower adiponectin levels on ICU admission would be associated with better survival, but this has been show in at least two studies [16], [20]. One hypothesis is that lower adiponectin acutely allows the proinflammatory response to predominate, but that as critical illness progresses into a more subacute or chronic phase, adiponectin rises or normalizes as the anti-inflammatory response becomes more important [36], and this biphasic response has been described by several authors [16], [18], [21], [36]. It is later in critical illness when production of anti-inflammatory cytokines such as IL-10 increases, and macrophages shift to their M2 form which has local anti-inflammatory and insulin-sensitizing features [36]. This biphasic theory of adiponectin in critical illness may partly explain why animal models have shown hypoadiponectinemia to be associated with worsened survival in sepsis and acute lung injury [6], [7]. Although not entirely analogous to human studies, these studies used adiponectin knockout mice, and thus the animals would not have the ability to increase adiponectin levels during the evolution of their critical illness.

Of the 10 ARDS case patients who were homozygous variant at rs2082940, only one died outside the first 2 weeks after onset of ARDS. This would suggest that patients generally died of multiorgan failure, overwhelming infection, and/or severe hypoxemic respiratory failure. Even the lone survivor had a prolonged hospital course lasting nearly 2 months from ARDS onset, and that case was the only underweight patient among the homozygous recessive group. The only salient features of nonsurvivors, as noted above, were a high percentage of BMI≥25 and sepsis/septic shock. Overall, this would support the hypothesis that blunting of the pro-inflammatory response in acute critical illness is detrimental, if indeed adiponectin levels are increased in these patients.

In one respect, we would not necessarily expect adiponectin to act differently in ARDS patients than in other critically ill subjects with acute inflammation of various etiologies. ARDS respresents an intense inflammatory syndrome localized in the lung but with systemic effects. Sepsis and septic shock also represent intense systemic inflammation, and such patients make up a large subset of our cohort developing ARDS. The observation that plasma cortisol and plasma adiponectin are highly correlated in healthy subjects may support that adiponectin is a marker for severity of illness [19], although we adjusted our results for APACHE III scores. In another respect, however, adiponectin may act differently in that obesity has been implicated as a risk factor for developing acute lung injury [37]–[39], yet diabetes has been well-described as a protective factor against developing ARDS [40]. Given adiponectin's link to both of these risk factors with quite opposite effects on ARDS susceptibility, there has been specific interest in adiponectin in ARDS [7].

We found no relationship between SNPs and BMI, and weak correlation between diabetes status and rs16861194. BMI is an imperfect measure in the ICU where weight can reflect the effects of acute illness or of fluid resuscitation. And although many studies have shown an inverse correlation between BMI and adiponectin, plasma levels are certainly affected by many genetic and nongenetic factors, and we examined only a small number of SNPs. BMI has also been associated with an increased risk of ARDS in a weight-dependent manner as mentioned above, [37] and BMI was significant in our multivariable models of outcomes.

We acknowledge limitations in this study. No measurements of adipokine levels are available for this cohort, and thus we cannot specifically link a genotype to a phenotypic correlate. We were also not able to control for insulin dosing or serum glucose levels throughout the ICU stay. This study is an exploratory analysis of available SNPs, several without known function. However, the most significant finding was in a SNP that has been studied, as above, although there were few homozygous recessive patients at this locus.

There are many strengths in this study. Perhaps most importantly, this is a large, prospective, well-characterized cohort with minimal misclassification. DNA quality was excellent with high genotyping success rates. This cohort also has a high percentage of patients with sepsis and septic shock, and along with ARDS itself, the cohort thus represents well the inflammatory sequelae of severe infection and critical illness. We controlled for diabetes and weight (BMI) as these are known to be associated with variations in adiponectin levels. This study also represents an addition to limited existing literature on the role of these genes and their polymorphisms in the critically ill.

## Conclusion

A common and potentially functional polymorphism in *ADIPOQ* may impact survival in ARDS. In general, adipokine-related genes may be relevant in survival from critical illness, possibly via insulin resistance and endothelial reactivity and inflammatory pathways.
